# The Microstructural Evolution, Tensile Properties, and Phase Hardness of a TiAl Alloy with a High Content of the β Phase

**DOI:** 10.3390/ma12172757

**Published:** 2019-08-28

**Authors:** Ning Cui, Qianqian Wu, Zhiyuan Yan, Haitao Zhou, Xiaopeng Wang

**Affiliations:** 1 School of Mechanical and Automotive Engineering, Qingdao University of Technology, Qingdao 266520, China; 2 Key Lab of Industrial Fluid Energy Conservation and Pollution Control, Ministry of Education, Qingdao University of Technology, Qingdao 266520, China; 3 Shanghai Spaceflight Precision Machinery Institute, Shanghai 201600, China; 4 State Key Laboratory of Advanced Welding and Joining, Harbin Institute of Technology, Harbin 150001, China

**Keywords:** TiAl alloys, β phase, hardness, tensile property

## Abstract

In this paper, the microstructure, deformability, tensile properties, and phase hardness of the Ti–43Al–2Cr–0.7Mo–0.1Y alloy with a high β phase content were investigated. Microstructural analysis showed that the β phase precipitated not only at the colony boundaries but also inside the lamellae due to its high content. A high-quality forging stock was prepared through one-step noncanned forging. The total deformation reached above 80%, suggesting that the alloy has good hot deformability compared to other TiAl alloys. The deformed microstructure was composed of fine and equiaxed grains due to dynamic recrystallization. The high β phase content was shown to contribute to the decomposition of the initial coarse lamellae. Tensile testing showed that the alloy has good room-temperature ductility, even if the β phase content reaches above 20%. This is inconsistent with a previous study that showed that a large amount of the hard β phase is detrimental to the room-temperature ductility of TiAl alloys. Nanoindentation testing showed that the hardness of the β phase in the current alloy is about 6.3 GPa, which is much lower than that in the Nb-containing TiAl alloys. Low hardness benefits the compatible deformation among various phases, which could be the main reason for the alloy’s good room-temperature ductility. Additionally, the influence of various β stabilizers on the hardness of the β phase was also studied. The β phase containing Nb had the highest hardness, whereas the β phase containing Cr had the lowest hardness.

## 1. Introduction

TiAl alloys are ideal candidates for use as components in the hot end of engines because of their attractive properties, such as their light weight and good high-temperature performance [1,2]. In the last two decades, studies on the composition design [3], microstructural control [4], and plasticity forming [5] of TiAl alloys have made considerable progress. Several TiAl parts have been preliminarily applied in the automotive and aviation industries [6,7]. However, their intrinsic brittleness has hindered their wider use. Thermal deformation is a suitable way to obtain a fine-grained microstructure, which is beneficial for the ductility of TiAl alloys [5,8]. Past research has confirmed that the hot workability of TiAl alloys can be significantly enhanced by the β phase with a disordered body-center cubic lattice at above 1150 °C [9,10]. The content of the β phase depends closely on β-stabilizers. Therefore, advanced β-solidifying TiAl alloys have become a focus of attention.

The β phase in β-solidifying TiAl alloys would transform into an ordered β_0_ phase at around 1100 °C [11,12]. For simplicity, both ordered and disordered phases are represented by β in this paper. Previous studies have shown that the β phase in Nb-containing TiAl alloys has a high hardness of 7–8 GPa, which is much higher than that of the γ and α_2_ phases [13]. The precipitation of the fine ω_0_ phase in the β phase caused by the high Nb content can further increase the hardness of the β phase (9–11 GPa) [14]. A high content of the β phase with high hardness is considered to be detrimental to the room-temperature (RT) ductility of TiAl alloys [15]. Therefore, research on Nb-containing TiAl alloys attempts to minimize the β phase content to ensure the hot deformability of TiAl alloys. On this basis, research has mainly concentrated on TiAl alloys with low β phase content. The β phase content generally ranges from 5% to 10% [16,17]. However, all these conclusions are based on the high hardness of the β phase in Nb-containing TiAl alloys. So far, very little research has been carried out on the phase hardness in TiAl alloys without Nb. It has not been confirmed whether the β phase also has such high hardness in TiAl alloys without Nb. Moreover, little information concerning TiAl alloys with a high content of the β phase is available. The influence of the high β phase content on the microstructure, deformability, and ductility of TiAl alloys is not clear.

In this paper, a Ti–43Al–2Cr–0.7Mo–0.1Y alloy with high content of the β phase was chosen as the research object. A fine-grained forging stock was produced by one-step noncanned deformation. The microstructure, tensile ductility, and phase hardness of the forging stock were investigated in detail. Additionally, the influence of various β stabilizers on the hardness of different phases in the TiAl alloy was studied for the first time.

## 2. Experimental Methods 

A Ti–43Al–2Cr–0.7Mo–0.1Y ingot was produced by using a vacuum consumable electric arc furnace. Several small specimens (∅8 mm × 12 mm) for hot compression tests and a large cylindrical specimen (∅70 × 90 mm) for hot forging were machined from the ingot. A high-quality forging stock was produced by noncanned forging with a reduction of above 80%. After forging, the alloy was annealed at 800 °C for 10 h to avoid cracking. In order to study the effect of β stabilizers on the hardness of the phases, several ingots of TiAl alloys with different β stabilizers were fabricated by using a vacuum nonconsumable arc furnace. The nanohardness of the various phases was measured using a Nano Indenter G200 equipped with a Berkovich diamond tip and an optical microscope (Agilent, PaloAlto, USA). The indentation depth is 60 μm. The maximum indentation force, the vertical resolution, and the force resolution of the instrument were 10 N, 0.01 nm, and 50 nN, respectively. The loading and unloading time was 10 s. The desired phase position could be observed using an optical microscope to ensure high measurement accuracy. The hardness value was obtained directly by the instrument. All indentations were further identified using a scanning electron microscope (SEM) after the measurement. Isothermal compression tests were carried out using a Gleeble 1500D simulator (DSI, Saint Paul, USA). Microstructures were examined by a Quanta 200F scanning electron microscope (SEM) (FEI, Hillsboro, USA) equipped with an electron backscattered diffraction (EBSD) system (FEI, Hillsboro, USA), and a Tecnai G2 F30 transmission electron microscope (TEM) (FEI, Hillsboro, USA). Energy-dispersive X-ray spectrometry (EDX) (FEI, Hillsboro, USA) and selected area electron diffraction (SAED) (FEI, Hillsboro, USA) were used to identify the phases. In order to avoid the effect of the deformed layer and residual stress, EBSD samples were produced by electropolishing technology. The samples were ground using 2000-grit SiC paper, followed by ultrasonic cleaning. Then the samples were electropolished in a solution of 60% methanol, 32% butanol, and 8% perchloric acid at −25 °C and 30 V. An acceleration voltage of 20 kV was used for EBSD image acquisition. The measured step size was 0.4 μm. The EBSD data were processed using orientation imaging microscopy (OIM) software, which provides a cleanup method to remove erroneous data. The TEM samples were produced using the ion milling method. The accelerating voltage and spatial resolution of the TEM system were 300 kV and 0.2 nm. Tensile tests were performed on an Instron universal testing machine (Boston, Massachusetts, USA).

## 3. Results and Discussion

### 3.1. The As-Cast Microstructure

**Figure 1:**
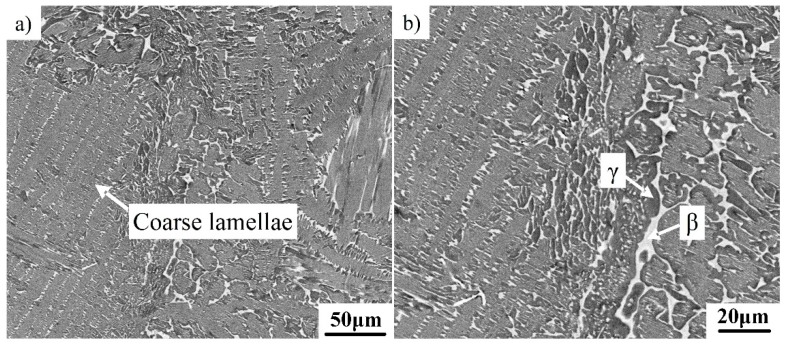
SEM micrograph showing the microstructure of the Ti–43Al–2Cr–0.7Mo–0.1Y ingot. (**a**) Coarse lamellae; (**b**) β and γ phases.

The microstructure of the cast Ti–43Al–2Cr–0.7Mo–0.1Y alloy was examined by SEM. The microstructure (Figure 1) mainly consisted of γ/α_2_ lamellar colonies, a bright β phase, and black γ phase, as indicated by the arrows. Compared with other β-solidifying TiAl alloys, the current alloy has more of the β phase [18]. Image analysis software shows that the β phase content was about 14%. The high β phase content could be ascribed to the strong β stability of Cr and Mo, which has been confirmed in the literature [3]. Previous studies on TiAl alloys with low β phase content have shown that the β phase mainly precipitates at colony boundaries [19]. Nearly no β phase can be observed inside the γ/α_2_ lamellae. By contrast, when the β phase content is high, it can be seen from Figure 1 that the β phase precipitates not only at colony boundaries, but also inside γ/α_2_ lamellae. Coarse γ/α_2_ lamellar colonies were divided by the β phase. Moreover, it can be seen that the distribution of the β phase in the microstructure is inhomogeneous. Coarse lamellar colonies and the unevenly distributed β phase were unfavorable to the mechanical properties of the TiAl alloys. Additional hot working is required to optimize the microstructure of the alloy.

### 3.2. Noncanned Forging and Microstructural Evolution

**Figure 2:**
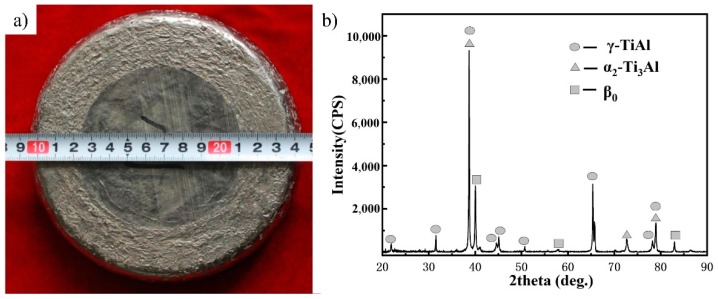
The appearance (**a**) and XRD pattern (**b**) of as-forged Ti–43Al–2Cr–0.7Mo–0.1Y alloy.

The hot deformability of TiAl alloys depends closely on their β phase content. In order to avoid cracking, TiAl alloys with low β phase content are generally deformed by means of isothermal forging or near-isothermal canned forging at 1200–1350 °C [9,20]. The deformation is generally set to 50–70%. Considering the high β phase content, the hot processing window of the current alloy should be wider than that of the TiAl alloys with low β phase content. According to the alloying design principle of β-containing TiAl alloys [3], TiAl alloys exhibit good plastic deformation properties when the deformation resistance is lower than 100 MPa under certain deformation conditions. Therefore, hot compression experiments were carried out to estimate the deformability of the current alloy. Results show that the deformation resistance is about 65 MPa when the Ti–43Al–2Cr–0.7Mo–0.1Y alloy was compressed at 1200 °C/0.05 s^−1^, suggesting that the alloy can be deformed under this condition. On this basis, a high-quality forging stock was prepared by one-step noncanned forging using hydraulic equipment (400 tons). The total deformation reached above 80%, and the appearance of the forging stock is shown in Figure 2a. The visual inspection shows no crack in the outer surface of the forging stock, which indicates that the Ti–43Al–2Cr–0.7Mo–0.1Y alloy has better hot deformability than other β-solidifying TiAl alloys with low β phase content. The XRD data show that the forging stock is composed of the γ, β, and α_2_ phases.

**Figure 3:**
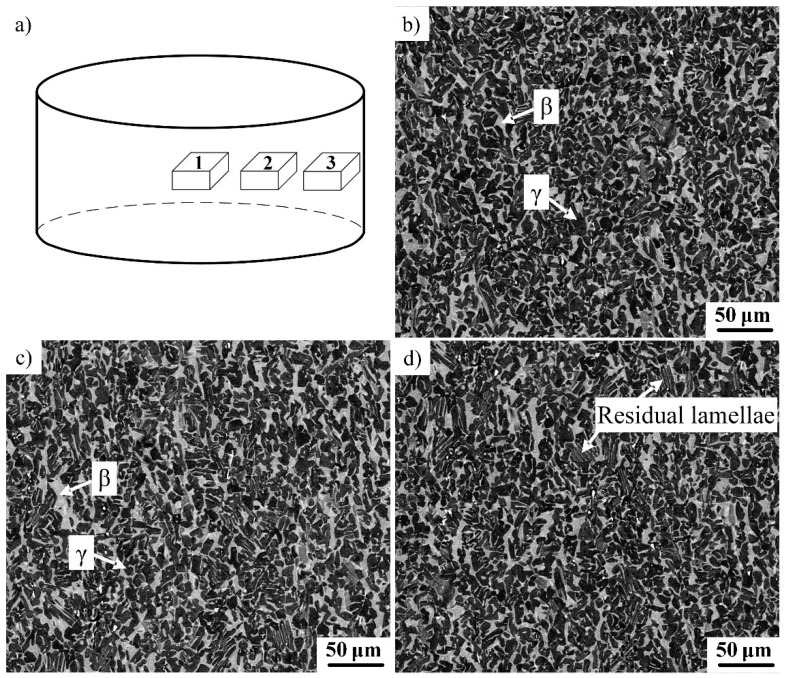
The microstructural characterization of the Ti–43Al–2Cr–0.7Mo–0.1Y billet. (**a**) Sampling position; (**b**) position 1; (**c**) position 2; and (**d**) position 3.

Compared with conventional β-solidifying TiAl alloys, the high β phase content in the current alloy inevitably influences the microstructural evolution of the alloys. In order to study the microstructural evolution during hot forging, the microstructure in different regions of the forging stock was observed by SEM, as shown in Figure 3. The sampling locations are indicated in Figure 3a. The microstructure in the center of the billet (position 1) is shown in Figure 3b. It can be seen that the microstructure mainly consists of a black γ phase and a white β phase. It is difficult to distinguish the α_2_ phase in the image due to its low content. The refined microstructure suggests that the initial coarse microstructure has been fully decomposed. Figure 3c shows the microstructure in the middle area of the billet (position 2), which is similar to that in the center area. Nearly no remaining lamellae can be found, indicating that the initial lamellae in the middle area were also decomposed. Figure 3d shows the microstructure on the edge of the billet (position 3), where it can be seen that most of the lamellae have decomposed. However, a closer examination reveals a few remaining lamellae, as indicated by the arrows. In spite of this, the current alloy still has high microstructural homogeneity. As reported in previous research, a large number of lamellae remain in the deformed microstructure, particularly at the edge of the forging stock [21,22]. This is because the decomposition of the initial lamellae depends closely on the deformation amount. A large deformation can provide enough driving force to cause microstructural evolution. The deformation amounts in different regions of the round forging stock are different. Maximum deformation generally appears at the center, while the deformation at the edge is minimal. For conventional TiAl alloys, deformation is difficult due to their poor hot deformability, which limits the decomposition of γ/α_2_ lamellae. By contrast, the current alloy has a high β phase content. Some of the soft β phase is distributed inside the γ/α_2_ lamellae, which can enhance the plastic deformation of lamellae around the β phase. This explains why only a few lamellae remain, even on the edge of the forging stock.

**Figure 4:**
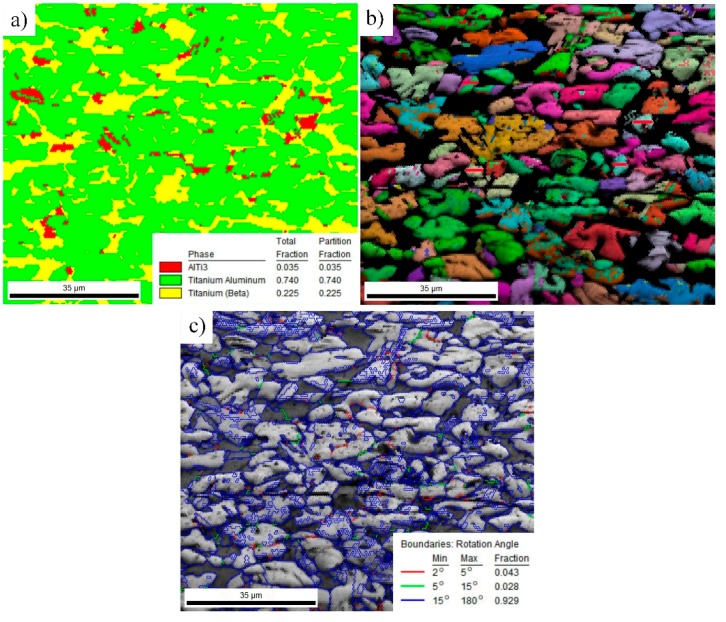
EBSD images showing the microstructure in the center of the forging stock. (**a**) Constituent phase; (**b**) inverse pole figure; (**c**) grain boundary.

In order to describe the as-forged microstructure more clearly, EBSD technology was employed to analyze the microstructure in the center area of the forging stock, as illustrated in Figure 4. The phase distribution features are shown in Figure 4a. The statistical results show that the content of the γ, β, and α_2_ phases are 74%, 22.5%, and 3.5%, respectively. The volume fraction of γ and α_2_ phases in the lamellar regions are generally measured by using TEM by orienting the γ and α_2_ platelets edge-on [23,24]. γ/α_2_ lamellae in TiAl alloys generally contain 80% γ lath and 20% α_2_ lath [23,24,25]. It is apparent that a great number of initial γ/α_2_ lamellae have been transformed into the γ phase during hot forging. A similar phase transformation has also been identified in previous studies on high-Nb-containing TiAl alloys [20]. The amount of the β phase increased slightly after forging. It can also be observed that the β phase was more uniformly distributed in the microstructure compared to the as-cast alloy. The inverse pole figure (Figure 4b) shows that both the β and γ grains have fine and homogeneous shapes. According to Figure 4b, the grain size measured by the manual measurement method is about 3–15 μm, indicating that severe plastic deformation can remarkably refine the microstructure of the current alloy. As shown in Figure 4c, the grain boundary (GB) feature of the as-forged microstructure was also detected. The data show that the fraction of the large-angle GB is above 90%, while the fraction of low-angle GB is 7.1%. Generally, the low-angle GB is a feature of substructures. The high density of large-angle GB indicates that dynamic recrystallization should be the main softening mechanism during the hot deformation of the current TiAl alloys.

**Figure 5:**
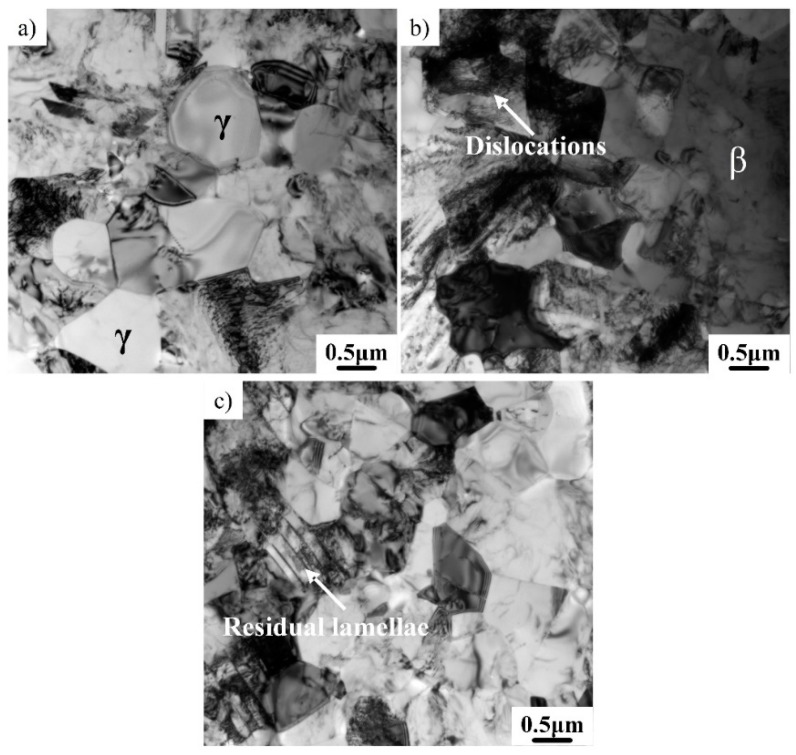
TEM brightfield image of the as-forged microstructure. (**a**) Globular γ; (**b**) irregular β; and (**c**) remaining lathes.

In order to further study the deformation mechanism, the features of the constituent phases in the deformed microstructure were also examined by TEM. As shown in Figure 5a, a great number of γ grains several microns in diameter can be identified. These γ grains have a low dislocation density and a relatively regular shape, indicating that these γ grains were formed by dynamic recrystallization [26]. Previous research has shown that the γ phase affords larger deformation compared to the α_2_ phase. The deformation of the γ phase can be realized by ordinary dislocations, superdislocations, and mechanical twinning, while the dislocation movement in the α_2_ phase is very difficult [27]. Dynamic recrystallization can significantly decrease the dislocation density of the γ phase. Some β phase was also identified, as shown in Figure 5b. It can be seen that the β phase has an irregular shape, indicating that dynamic recrystallization has not occurred for the β phase. The β phase has a body-centered cubic structure and higher stacking fault energy [28]. Dynamic recovery is more likely to occur for the β phase [29]. It can also be seen that the region around the β phase has a high dislocation density. The soft β phase has excellent deformability and can contribute to the deformation of alloys. Moreover, a small quantity of the remaining lamellae can also be found in the deformed microstructure, as shown in Figure 5c. This is because the decomposition of the initial γ/α_2_ lamellae is related to the relative orientation between the lamellae and loading [30].

### 3.3. Tensile Properties

**Figure 6:**
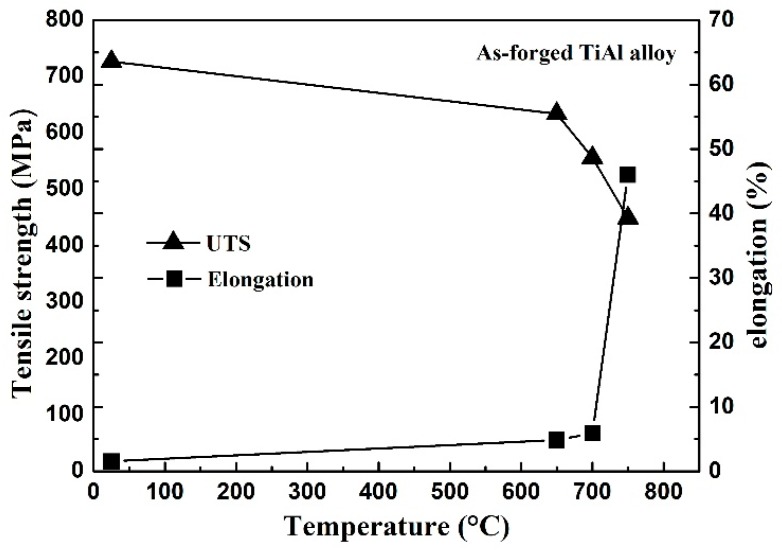
The dependence of tensile strength and elongation on the temperature.

In order to study the effects of high β phase content on tensile properties, tensile tests were carried out on the Ti–43Al–2Cr–0.7Mo–0.1Y alloy. The dependence of strength and elongation on temperature is summarized in Figure 6. The RT tensile strength is about 726 MPa, which is higher than that of the Cr- and Mn-containing TiAl alloys [31], but lower than that of high-Nb-containing TiAl alloys [32]. The RT elongation is about 1.5%, which is close to that of the TiAl alloys with low β phase content. Thus, the current forging stock has good RT tensile properties, even though the β phase content reached about 20%. A similar phenomenon was also observed in a study on the Ti–43Al–9V–Y alloy. Kong et al. found that the RT elongation of a Ti–43Al–9V–Y alloy with about 20% β phase can also reach about 2%. This is inconsistent with the conclusion on the Nb-containing TiAl alloys that high β phase content can significantly reduce the ductility of TiAl alloys [33]. The reason for this phenomenon is discussed in Section 3.4. The tensile properties of the alloy at 650–750 °C were also tested. At 650 °C, the tensile strength and the elongation are 634 MPa and 4.8%, respectively. When the testing temperature is 700 °C, the alloy still has high strength (556 MPa) and superior ductility (5.9%). However, as the temperature is raised to 750 °C, the alloy suddenly has very high elongation and very low strength, suggesting that 750 °C is higher than the ductile-brittle transition temperature of the alloy. Thus, the working temperature of a Ti–43Al–2Cr–0.7Mo–0.1Y alloy should be not higher than 700 °C.

### 3.4. Nanohardness of the Constituent Phases

**Figure 7:**
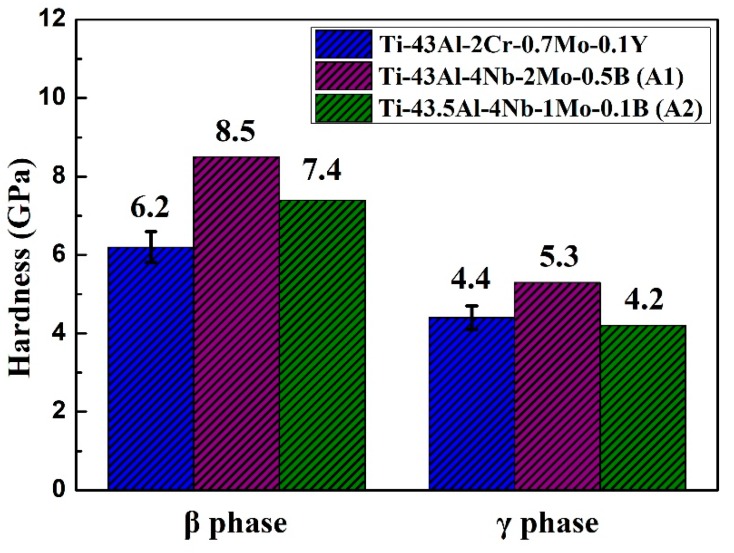
Nanohardness of constituent phases in the Ti–43Al–2Cr–0.7Mo–0.1Y alloy.

Previous studies on Nb-containing TiAl alloys showed that the hardness and brittleness of the β phase are very detrimental to the RT ductility of alloys [22]. The elongation of TiAl alloys with high β phase content is generally very low, which is inconsistent with the current study. In order to clarify this issue, nanoindentation tests were conducted on the Ti–43Al–2Cr–0.7Mo–0.1Y alloy. Three indentations were performed on each phase to ensure measurement accuracy. In this study, we only measured the hardness of the γ and β phases. It is hard to measure the hardness of the α_2_ phase due to its low content in the microstructure. Figure 7 shows a comparison of the phase hardness in different alloys, including the current alloy, A1, and A2 [13,15]. As shown in Figure 7, the hardness value of the γ phase in the current alloy is 4.4 GPa, which is close to that (4.2 GPa) in the A2 alloy and slightly lower than that (5.3 GPa) in the A1 alloy. By contrast, the β phase in the current alloy has a hardness value of 6.3 GPa, which is much lower than that in the A1 alloy (8.5 GPa) and that in the A2 alloy (7.4 GPa). It can also be seen that the hardness difference between the γ and β phases is reduced. The low hardness of the β phase could help coordinate the deformation of various phases at RT, thereby decreasing harm to the RT ductility of TiAl alloys. This also explains why the Ti–43Al–2Cr–0.7Mo–0.1Y alloy with high β phase content still has superior tensile properties. Moreover, it is obvious that the hardness of the various phases in TiAl alloys is related to β-stabilizers.

**Figure 8:**
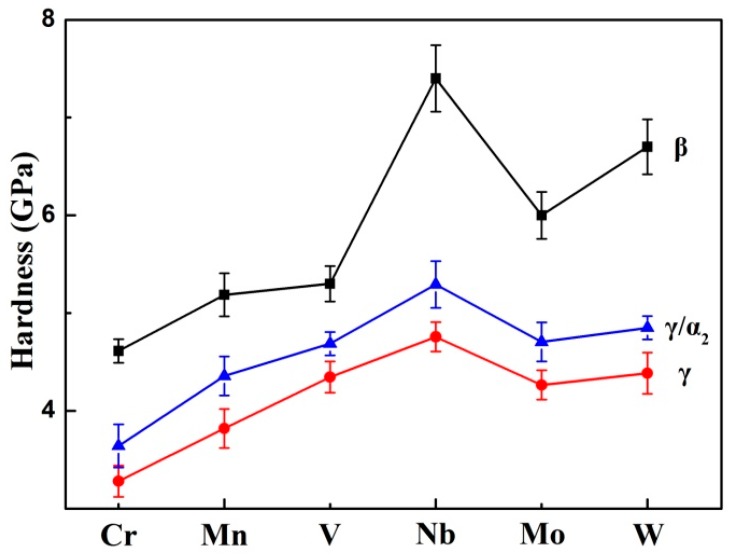
The effect of β stabilizers on the hardness of constituent phases in TiAl alloys.

In order to further study the effect of β–stabilizers on the phase hardness, nanoindentation tests were performed on the β phase, γ/α_2_ lamellae, and γ phase in various TiAl alloys containing different β-stabilizers. To investigate the phase hardness more accurately, the β phase content was controlled at 3–10 vol % in this study. Previous studies have confirmed that different elements exhibit different β stability [3]. The amounts of different elements required to introduce β phase mainly depend on their β stability. Thus, several alloys with nominal compositions of Ti–43Al–10Nb, Ti–43Al–1.5Mo, Ti–43Al–1.5W, Ti–43Al–5Cr, Ti–43Al–5Mn, and Ti–43Al–6V were chosen as research objects. All of these alloys have similar β-solidifying microstructures, which consist of γ/α_2_ lamellae, as well as β and γ phases. The detailed microstructures have been reported in other studies [3,34]. Figure 8 shows the average hardness values of the constituent phases in various TiAl alloys. It can be seen that the hardness of the constituent phases depends closely on the β-stabilizers. As shown in Figure 8, the hardness of the γ phases in different TiAl alloys is between 3.5 GPa and 4.7 GPa, and the hardness of the γ/α_2_ lamellae in different TiAl alloys is between 3.6 GPa and 5.2 GPa. This indicates that the hardness of the γ phases and γ/α_2_ lamellae are less affected by β-stabilizers. By contrast, the hardness of the β phase is more affected by β-stabilizers. The β phase introduced by adding Nb exhibits the highest hardness (7.4 GPa). This hardness value is in good agreement with other results published in previous studies [13,15]. The β phase with high hardness is detrimental to the RT ductility of TiAl alloys. The β phase introduced by adding Cr exhibits the lowest hardness (4.6 GPa). Low hardness could help improve the RT ductility of TiAl alloys. This provides a new idea for the composition design of β-solidifying TiAl alloys with excellent ductility. Further research on this aspect will be conducted in the future.

## 4. Conclusions

(1) The Ti–43Al–2Cr–0.7Mo–0.1Y alloy with a high β phase content has a coarse and inhomogeneous as-cast microstructure. The β phase precipitates not only at the lamellar boundaries, but also inside the lamellae. The Ti–43Al–2Cr–0.7Mo–0.1Y alloy has good hot workability due to the high content of its β phase. A high-quality forging stock was prepared after one-step forging with 80% deformation. A high β phase content contributes to the decomposition of the coarse as-cast microstructure during hot forging.
(2) The forging stock has a uniform and fine microstructure, which is composed of γ and β phases and a small amount of the α_2_ phase. The alloy has good room-temperature ductility, even though it contains a high β phase content. The room-temperature elongation can reach about 1.5%, which can be ascribed to the low β phase hardness. The nanohardness of the β phase in the current alloy is about 6.3 GPa, which is much lower than that in the high-Nb-containing TiAl alloys.
(3) The hardness of the β phase depends closely on β-stabilizers. The β phase containing Cr, Mn, or V has low hardness, while the β phase containing Nb, Mo, or W has high hardness. Reducing the hardness of the β phase by alloying may be an effective way to improve the ductility of β-solidifying TiAl alloys.
